# Prenatal diagnosis in a fetuses with a clenched hands, overlapping fingers, and clubfoot due to MED12 deficiency in three affected siblings: A case report

**DOI:** 10.3389/fgene.2023.1037345

**Published:** 2023-07-12

**Authors:** Huiqin Xue, Qiaoyin Tang, Yu Feng, Chenyue Zhao, Ke Xu, Weiyue Gu, Zhaoyu Xue, Xinyan Li, Jinsong Jiang, Hongyong Lu, Xiayu Sun, Jianrui Wu, Guizhi Cao

**Affiliations:** ^1^ Department of Cytogenetic Laboratory, Children’s Hospital of Shanxi, Women Health Center of Shanxi, Affiliated Hospital of Shanxi Medical University, Taiyuan, China; ^2^ Department of Paediatric Medicine, Shanxi Medical University, Taiyuan, China; ^3^ Beijing Chigene Translational Medicine Research Center Co., Ltd., Beijing, China; ^4^ Department of Obstetrics and Gynecology, Children’s Hospital of Shanxi, Women Health Center of Shanxi, Affiliated Hospital of Shanxi Medical University, Taiyuan, China

**Keywords:** MED12, clenched hand, overlapping fingers, clubfoot, case report

## Abstract

A fetal clenched hand with overlapping fingers is more common in aneuploidy syndrome and was not well-documented in MED12 deficiency. This study reports the clinical and genetic findings of three affected siblings from a Chinese family. The chromosome karyotype analysis diagram shows that karyotypes of the three children were normal. Trio whole-exome sequencing and Sanger sequencing verification found that there was a MED12 R296Q variant in normal mothers and their two offspring. A pattern of clenched hand with overlapping fingers (clinodactyly) and clubfoot was found in all the three affected siblings by three-dimensional ultrasound. The discovery of this case shows that even if the chromosome karyotype is normal, comprehensive prenatal genetic diagnosis is required when the ultrasound results show a clenched hand with clinodactyly and clubfoot symptoms.

## Introduction

Pathogenic variants in the *MED12* gene cause multiple X-linked recessive disorders including FG syndrome type 1 (FGS1, MIM: #305450), Lujan syndrome (LS, MIM: #309520), X-linked Ohdo syndrome (XLOS, MIM: #300895), and non-specific intellectual disability (NSID) (Lyons, 1993). Plassche and Brouwer (2021) recently reviewed 77 *MED12* variants, and they found that the causative missense variants were not clustered in one specific protein region, which did not lead to a clear genotype–phenotype association. Furthermore, with the exception of NSID, these disorders have similar phenotypes of congenital deformities, e.g., macrocephaly, mandibular retrognathia, musculoskeletal anomalies, clinodactyly, and heart defects, that could, theoretically, be identified by 2D or 3D prenatal sonography (Lyons, 1993; Patil et al., 2017; Amodeo et al., 2020).

Overlapping fetus fingers is a prenatal ultrasound observation commonly found in fetuses with malformation syndrome and aneuploidy disorders, e.g., trisomies 18, 13, and 21 (Linder et al., 2009; Schumacher et al., 2010). Typically, the second finger is seen to overlap the third one when there was a concurrent clenched fetal hand (Boyd, 2003). In the case of aneuploidy, prenatal screening tests, such as non-invasive prenatal testing (NIPT) and invasive karyotyping, are capable to determine the genetic causes (Gregg et al., 2016).

In this study, we first identified the maternally inherited recurrent variant MED12 R296Q in an aborted fetus, whose phenotype and genetic test results suggested a diagnosis of MED12 deficiency in the first child who was deceased prematurely. We subsequently performed a prenatal genetic diagnosis on the third child of this family and found the same recurrent variant and fetal sonographic characteristics.

## Case presentation

The family consisted of eight members of three generations, where all the offspring of the third generation represented inborn or intrauterine abnormalities; so, an induced abortion was performed on the proband (Figure 3A). The mother of the proband was 33 years old, and she was healthy. No family history of other inherited disorders was announced. The adult subjects gave informed consent on behalf of the family members enrolled in this study.

**FIGURE 1:**
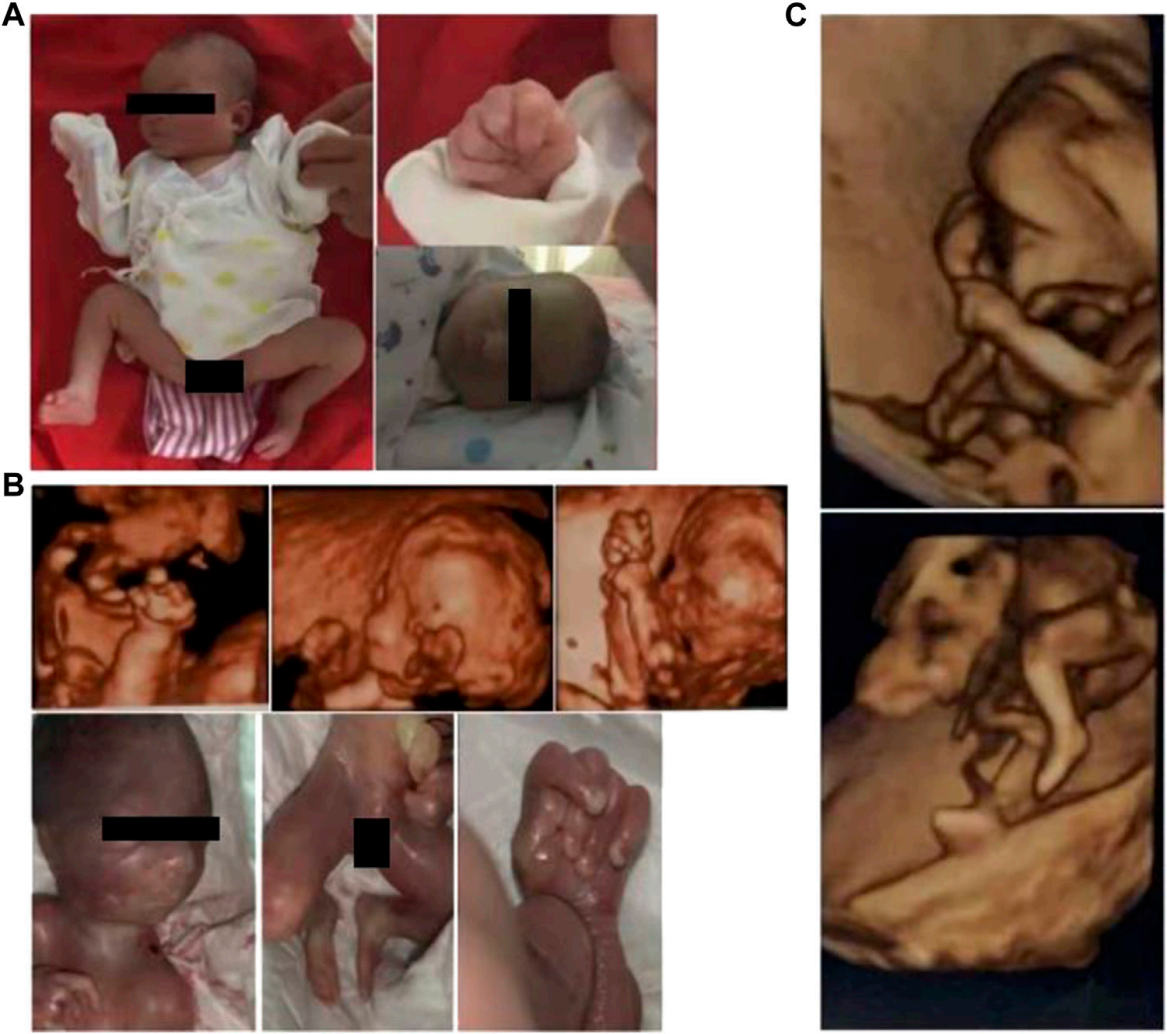
Photographs or 3D sonographs of the three affected siblings, denoted by **(A–C)** from old to young, respectively. All the children had clenched hand with overlapping fingers and clubfoot (was difficult to be further classified). It is worth noting that microreflexia was found in the oldest patient and the second child **(A, B)**, which cannot be recognized in the third child **(C)** due to the small gestational age.

The couple came to our hospital in October 2019 because of an abnormal prenatal ultrasound of their second child and considering that their first child, male, experienced an overdue birth (born by caesarean section at 42 weeks of gestation) and inborn abnormalities. In the third trimester, a prenatal ultrasound showed polyhydramnios and intrauterine growth retardation in their first child. After his birth, the boy had feeding difficulty, a congenital clenched hand with overlapping fingers, and clubfeet with broad halluces (hallux rigidus); so, the child was hospitalized for about 10 days. Because of multiple deformities, the child’s parents gave up the treatment. The child was discharged from the hospital and then died at 3 months of age because of poor feeding (Figure 1A). Physical examination showed the patient had micrognathia, limbs hypertonia, and hyporeflexia. A hearing test could not be accomplished due to his non-cooperation. Color Doppler found that he had congenital heart disease (patent foramen ovale and patent ductus arteriosus) and a separation in the collecting system in the left kidney. His brain MRI showed long T1W1 signal on the bilateral globus pallidus. Both the karyotyping and genetic screening for metabolic disorders were normal.

In the second child, the proband, prenatal 3D-US showed polyhydramnios, micrognathia, clenched fetal hand with overlapping fingers, and clubfoot at a gestation period of 22 weeks, where the extremity anomalies were similar to the findings in the first child (Figure 1B).

In March 2022, the couple came to our hospital again because of abnormal prenatal tests in the third baby. At week 12 of gestation, color Doppler showed thickened nuchal translucency (NT) in the fetus. Similarly, prenatal 3D-US showed the fetus had a clenched fetal hand with overlapping fingers and clubfoot (Figure 1C).

## Materials and methods

### Cytogenetic karyotype

A measure of 2 mL peripheral blood was collected and cultured in suspension for 3 days. Then, 20 mL amniotic fluid was collected and cultured in Petri dishes for 8 days. The preparations were conducted according to the standard procedures (GTG-banding). Twenty metaphases from independent colonies were counted, five of which were karyotyped. The International System for Human Cytogenomic Nomenclature (ISCN 2020) was employed to describe the karyotypes.

### Genetic tests

Peripheral blood, umbilical cord blood, or amniotic fluid of the subjects was collected and sent to Chigene (Chigene Ltd., Beijing, China) for all the genetic tests. Trio whole-exome sequencing (WES) was performed in the proband and his parents, using xGen Exome Research Panel v1.0 (IDT, Iowa, United States) for library construction, and high-throughput sequencing was performed using the NovaSeq 6000 sequencer (Illumina, San Diego, United States). The paired-end reads were called using the Burrows–Wheeler Aligner (BWA) software package and then aligned to the Ensemble GRCh37/hg19 reference genome. The single-nucleotide variant and indel shorter than or equal to 50 bp were called using GATK. Using the Chigene analysis system for inherited disorders (https://cloud.chigene.org/), all the yielded variants were classified according to the American College of Medical Genetics (ACMG) clinical practice guidelines. To perform Sanger sequencing for genetic screening in the fetus (III-3) and the other family members, primers F-GTAGGATACAGCTGAGGCCAAAA and R-CTTCTCCGAGTAAGGCT-TGGAAT were constructed for PCR.

## Results

**FIGURE 2:**
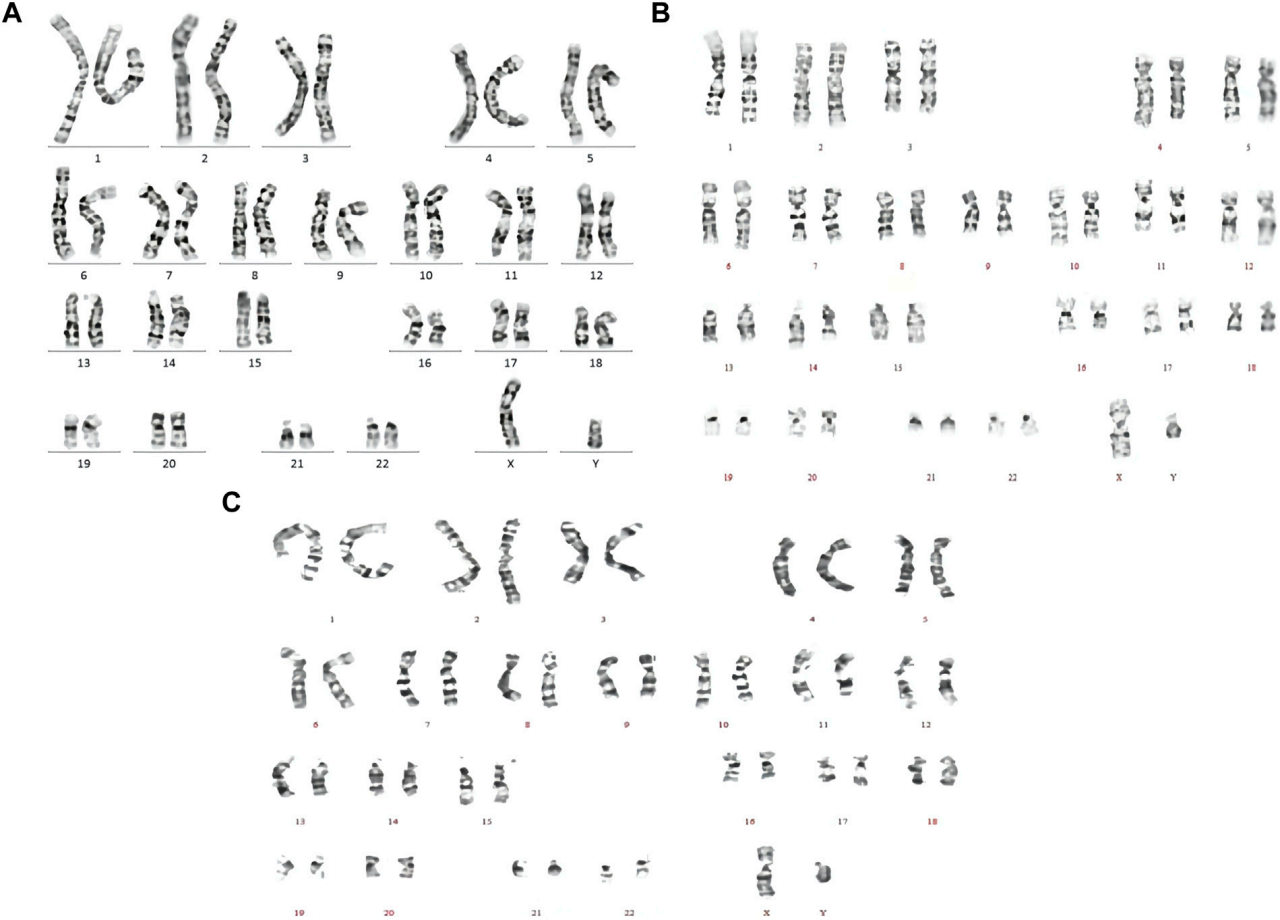
Chromosome karyotype analysis diagrams of the three affected siblings, denoted by **(A–C)** from old to young, respectively. Karyotyping of all the children was normal.

The chromosome karyotype analysis diagram shows that karyotypes of the three children were normal (Figures 2A–C).

**FIGURE 3:**
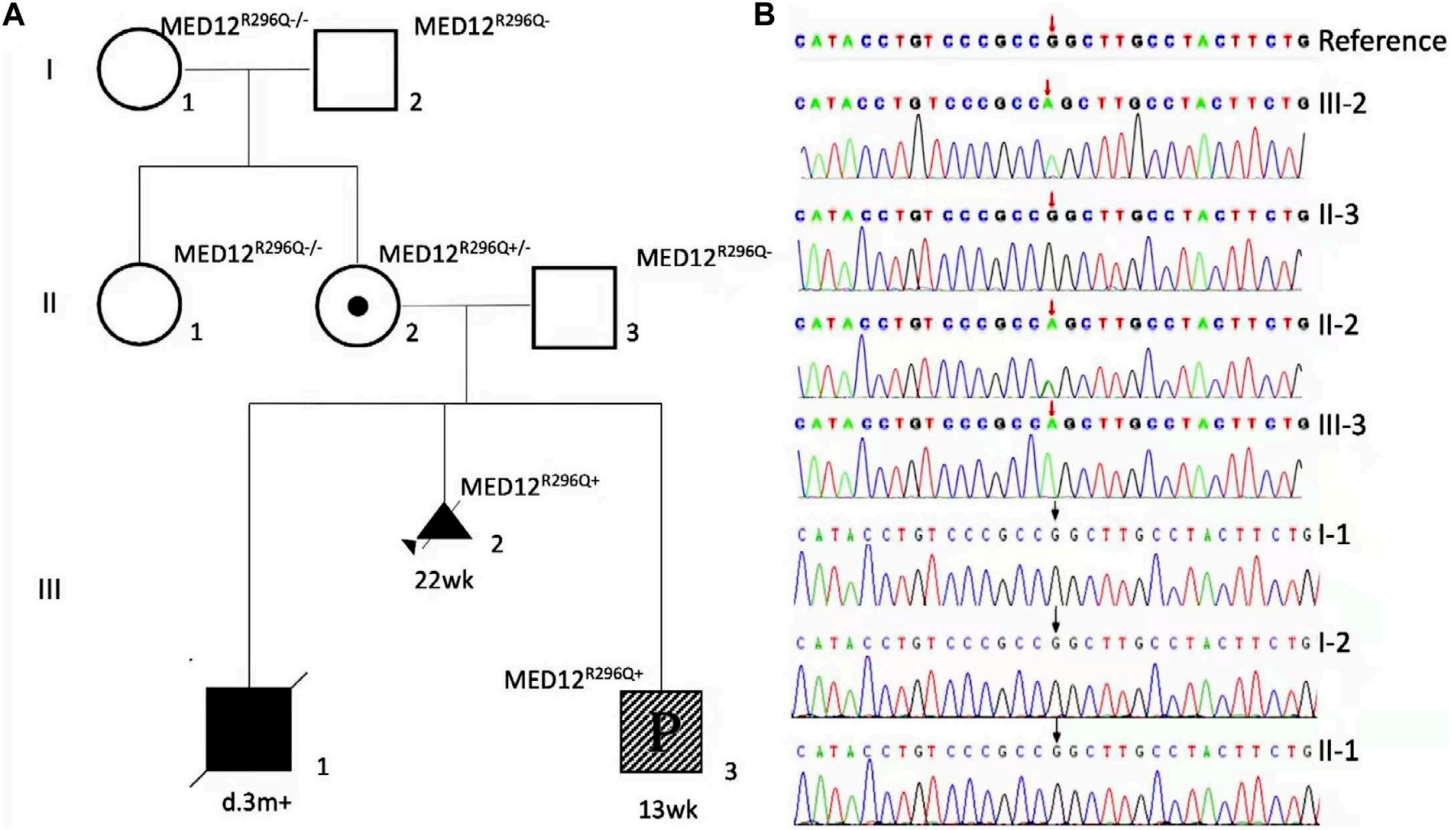
Family is X-linked recessive inheritance (XR), with square representing male and circle representing female. Ⅰ-1, Ⅰ-2, Ⅱ-1, and Ⅱ-3 are wild type, and III-2 is a family proband detected by WES that the diseased fetus was aborted at the gestational age of 22 weeks. II-2 is an asymptomatic carrier (the mother of the proband), III-3 was found to be sick at the gestational age of 13 weeks. Ⅲ-1 died at 3 months, and the child did not undergo genetic testing **(A)**. Pedigree diagram of the affected family with the members tested by WES or MED12 (NM_005120.2) c.887G-targeted sequencing. Sanger sequencing detected NM_005120.2) c.887G>A in three of the eight family members **(B)**.

Trio WES showed hemizygous *MED12* (NM_005120.2): c.887G>A, p. R296Q, in the proband (Ⅲ-2), and the mother is a carrier (confirmed by Sanger sequencing, Figure 3B). The MED12 variant R296Q was previously reported in patients with XLOS or NSID, and it is pathogenic, according to the ACMG clinical practice guidelines. Using copy number variant (CNV) analysis based on the WES data, we found no causative CNVs or aneuploidy in the proband and the parents. The CNV results were subsequently verified by copy number variation sequencing (CNV-seq).

Genetic screening of MED12 R296Q was negative in the proband’s grandparents and his aunt, indicating it was a *de novo* incident in the mother (Figures 3A,B). In the third child, the test of MED12 R296Q was positive, and it was hemizygous, showing the child was a male (III-3, Figure 3B).

## Discussion

MED12 deficiency is a rare condition which includes the phenotypes of FG syndrome type 1 (FGS1, MIM: #305450), Lujan syndrome (LS, MIM: #309520), X-linked Ohdo syndrome (XLOS, MIM: #300895), non-specific intellectual disability (NSID), and Hardikar syndrome (HS, MIM: #301068) (Lyons, 1993). In addition to HS, a clinical syndrome that describes abnormalities in carrier females, the other four syndromes all cause developmental and/or intellectual disabilities in affected males (Lyons, 1993). Detailed differentiation of the wide variety of phenotypes due to these MED12 hemizygous variants helps differential diagnosis in postnatal patients. However, known clues to MED12 deficiency by prenatal tests are scarce. By prenatal sonography, agenesis of the corpus callosum (ACC) (Jiang et al., 2019), cleft lip and cleft palate (Faergeman et al., 2021), microphthalmia (Amodeo et al., 2020; Faergeman et al., 2021), microretrognathia (Amodeo et al., 2020), heart disease (Amodeo et al., 2020; Faergeman et al., 2021), broad thumbs and halluces (Kato et al., 1994; Graham et al., 1999), and fetal digital pads (Clark et al., 2009) have been reported in fetal MED12 deficiency; in terms of phenotype, congenital clinodactyly and overriding toes were documented in some of postnatal patients with XLOS (Patil et al., 2017), which indicates possible prenatal ultrasound findings. In the oldest patient in this study, the postnatal findings of hallux rigidus and congenital heart disease (CHD) prompt a diagnosis of MED12 deficiency, although the patient failed to receive a comprehensive genetic test. It is worth noting that a review of the karyotype of the oldest patient provides a hypothetical scenario that couples may prefer to continue pregnancy when prenatal screening results, such as clenched hand and overlapping fingers, could not be explained to accomplish a diagnosis.

In fetal extremity anomalies, overlapping fingers/clinodactyly with or without clenched hand is remarkable in early (12–13 weeks) gestation screening (Bronshtein et al., 1995), which makes it a more favorable indicator than facial deformities such as microphthalmia or microretrognathia in fetuses with MED12 deficiency (Caro-Llopis et al., 2016; Amodeo et al., 2020). In the case of the third child in this study, it was difficult to specify whether there was a microretrognathia based on 3D sonography in early gestation (Figure 1C). Revelation from this case is that the curled posture of fetuses typically makes facial features difficult to be identified, while the limbs features are more likely to be detected by ultrasound because they are commonly located on the surface.

Based on the study in 77 causative MED12 variants, Plassche and Brouwer (2021) declared that the three clinically named syndromes, FGS1, LS, and XLOS, were attributed to only eight out of 77 variants, which means a high heterogeneity of phenotype of MED12 deficiency. This conclusion was supported by our and previously reported four cases (Caro-Llopis et al., 2016; Martínez et al., 2017; Rocchetti et al., 2021) due to MED12 R296Q. Rocchetti et al. (2021) compared the phenotypes in the four patients with R296Q, who shared only microcephaly/brachycephaly and micrognathia/microretrognathia out of 24 identified craniofacial signs, and the only phenotype, broad thumbs and halluces, of extremities was found in one of the patients. In comparison with previously reported cases due to MED12 R296Q (Caro-Llopis et al., 2016; Martínez et al., 2017; Rocchetti et al., 2021) and clinical features summarized by Plassche and Brouwer (2021), the pattern of fetal clenched hand with overlapping fingers and clubfoot is reported for the first time in our study. However, among the types of fetal hand and foot abnormalities, overlapping fetus fingers are characteristic phenotypes of fetuses carrying aneuploidy, such as trisomies 18, 13, and 21 (Witters et al., 2011; Rosa et al., 2012), which are usually diagnosed by prenatal chromosome screening. Clinicians are often unable to provide clear decision-making advice when chromosome results are unclear, such as low-risk NIPT results or karyotype analysis is negative. We cannot exclude other etiologies of the unique finding besides aneuploidy, and it could, for example, be caused by other variants having linkage disequilibrium with MED12 R296 and causing phenotypes similar to MED12 deficiency. So, further findings of the fetal phenotype in unrelated cases due to MED12 variants are expected.

In this study, we focused on the consequences for this affected family. First, in the absence of a similar family history, the couple chose to give birth to their first child with an abnormal prenatal examination. It is very likely that routine genetic screening such as karyotyping did not determine a diagnosis, which might bring a fluke mind to both the doctors and the family. Second, based on a solid prenatal diagnosis for the second child, the personalized genetic screening produced benefits in the follow-up, and induced abortion, subsequently, was performed on the third child. Based on the findings in this study, the family had their healthy fourth child by a pre-implantation genetic diagnosis. After three failed pregnancies, the mother accepted our advice and had a healthy child through pre-implantation genetic diagnosis. The mother was very satisfied with the treatment and results.

Through this case, our study raises an important decision-making question for prenatal screening: the aneuploidy syndrome-like phenotype in fetus with MED12 deficiency has never been focused on, and our study facilitated the prenatal diagnosis of the disease and expanded the phenotypic spectrum. Second, even if the diagnostic criterion was certain, a comprehensive prenatal diagnosis technique should, as early as possible, be considered when karyotyping does not provide a positive result for a fetus with clenched hands, overlapping fingers, and clubfoot, which is usually thought to be attributed to aneuploidy syndrome.

## Data Availability

The datasets for this article are not publicly available due to concerns regarding participant/patient anonymity. Requests to access the datasets should be directed to the corresponding author.
